# Topical Delivery of Protein and Peptide Using Novel Cell Penetrating Peptide IMT-P8

**DOI:** 10.1038/srep26278

**Published:** 2016-05-18

**Authors:** Ankur Gautam, Jagpreet Singh Nanda, Jesse S. Samuel, Manisha Kumari, Priyanka Priyanka, Gursimran Bedi, Samir K. Nath, Garima Mittal, Neeraj Khatri, Gajendra Pal Singh Raghava

**Affiliations:** 1 Bioinformatics Centre, CSIR-Institute of Microbial Technology, Chandigarh-160036, India; 2 Department of Protein Science and Engineering, CSIR-Institute of Microbial Technology, Chandigarh-160036, India; 3 Experimental Animal Facility, CSIR-Institute of Microbial Technology, Chandigarh-160036, India

## Abstract

Skin, being the largest organ of the body, is an important site for drug administration. However, most of the drugs have poor permeability and thus drug delivery through the skin is very challenging. In this study, we examined the transdermal delivery capability of IMT-P8, a novel cell-penetrating peptide. We generated IMT-P8-GFP and IMT-P8-KLA fusion constructs and evaluated their internalization into mouse skin after topical application. Our results demonstrate that IMT-P8 is capable of transporting green fluorescent protein (GFP) and proapoptotic peptide, KLA into the skin and also in different cell lines. Interestingly, uptake of IMT-P8-GFP was considerably higher than TAT-GFP in HeLa cells. After internalization, IMT-P8-KLA got localized to the mitochondria and caused significant cell death in HeLa cells signifying an intact biological activity. Further *in vivo* skin penetration experiments revealed that after topical application, IMT-P8 penetrated the stratum corneum, entered into the viable epidermis and accumulated inside the hair follicles. In addition, both IMT-P8-KLA and IMT-P8-GFP internalized into the hair follicles and dermal tissue of the skin following topical application. These results suggested that IMT-P8 could be a potential candidate to be used as a topical delivery vehicle for various cosmetic and skin disease applications.

Delivery of therapeutic molecules through the skin is gaining tremendous scientific attention over the years. Though delivery of therapeutics through the skin is non-invasive, simple and provides a lot of benefits to patients, it is very challenging as the skin provides a protective barrier against external influences^1^. The outermost layer of skin, stratum corneum (SC) is made up of keratin-filled, non-viable cells embedded in a crystalline intercellular lipid domain^2^ and impermeable to almost all compounds and drugs having a molecular weight of more than 500 Da^3^. Therefore, attempts have been made in the past to examine the innovative novel methods to increase the permeability of skin^4^,^5^,^6^,^7^,^8^,^9^,^10^. Skin delivery is mainly focused either on topical delivery to treat local skin conditions or transdermal drug delivery, which involves the delivery of drugs through skin layers into systemic circulation^4^. Topical delivery offers several advantages over the conventional dosage forms (oral and intravenous) like avoidance of first-pass metabolism, ease of application, reduces the enzymatic degradation associated with oral delivery, improved patient compliance, and controlled release of drugs^4^,^11^.

Recently, peptides have been emerged as safer alternatives to enhance the delivery of small and large molecules into and across the skin^12^. In this context, transdermal delivery of therapeutics using cell-penetrating peptides (CPPs) is an attractive and novel approach^11^. CPPs constitute a family of small peptides, which have an inherent ability to traverse biological membrane without causing significant membrane damage^13^,^14^,^15^. Therefore, in the past, CPPs have been used widely for the intracellular delivery of many therapeutic molecules such as siRNA^16^,^17^, protein^18^, and peptides^19^ *in vitro* and *in vivo*. However, use of peptides as the transdermal delivery system is new^12^ and still in its infancy. In the recent past, a few studies have shown that small peptides such as TAT^20^, polyarginine^21^, meganin^22^, penetratin^23^, TD-1^24^ and SPACE-peptide^25^ can penetrate the skin’s protective barriers to get to the deeper layers and enhance the transdermal delivery of various therapeutic molecules like siRNA^26^, cyclosporine A^27^, insulin, etc. The results of these studies are promising and raise hope for CPPs to be used as a transdermal delivery system to combat skin diseases.

We recently identified and characterized a novel human protein-derived arginine-rich CPP, IMT-P8 (IMT-P8; Institute of Microbial Technology-Peptide 8)^28^ using *in silico* prediction algorithm, CellPPD^29^ and followed by experimental validation. The aim of the present study was to investigate the cargo delivery capability of IMT-P8 in different model systems, including topical delivery. Because IMT-P8 is a novel CPP and its cargo delivery potential was not explored previously, first we have examined the type of cargo IMT-P8 could deliver. Before performing experiments on mouse skin for topical delivery, we first examined the delivery of cargoes using IMT-P8 on continuously growing human cells. These *in vitro* experiments confirmed that IMT-P8 is capable of delivering cargoes (FITC, peptide and protein) into variety of human cells. Next, we sought to determine whether IMT-P8, which possesses the ability to cross the plasma membrane barrier of human cells, could also cross the stratum corneum the outermost dead cell layer of skin and deliver cargoes into the skin layers. Many CPPs *i.e.* TAT^30^, penetratin^31^ and oligoariginine^32^ have been reported previously to deliver cargoes (nucleic acid, protein, peptides, *etc*) in different cell lines. Later, several studies demonstrated the skin penetrating efficiency of these CPPs and cargo complexes on laboratory animal skin^21^,^33^,^34^,^35^. Therefore, in the present study, we have also evaluated the cargo delivery potential of IMT-P8 in different model systems.

We constructed IMT-P8-GFP fusion protein and IMT-P8-KLA fusion peptide to investigate the delivery of GFP and proapoptotic peptide, KLA into mouse skin as well as in various cancer cell lines. Our results demonstrate that IMT-P8 facilitates the delivery of GFP and KLA into mouse skin and in different human tumor cells at very low concentration suggesting that IMT-P8 might be beneficial for the treatment of local skin infections and various cosmetic applications.

## Results

### *In vitro* delivery of KLA by IMT-P8

**Figure 1:**
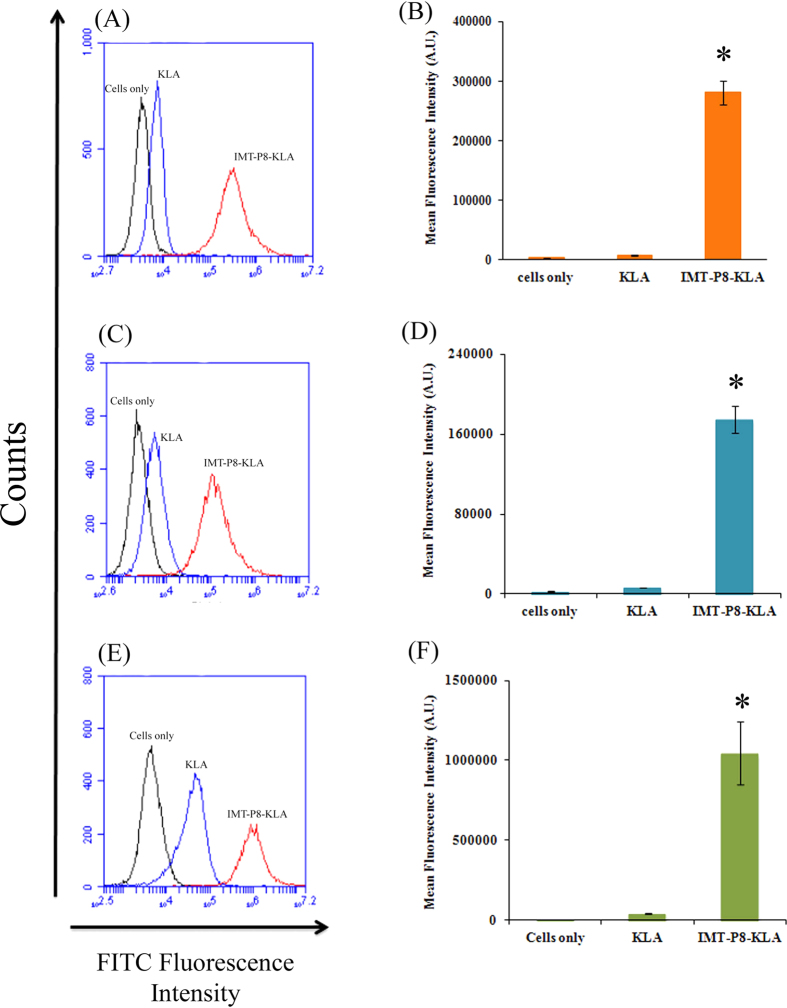
Cellular uptake of KLA and IMT-P8-KLA as determined by FACS analysis. Frequency distributions of FITC fluorescence intensity in (**A**) HeLa, (**C**) MDA-MB-231 and (**E**) PC3 cells incubated with FITC labeled KLA and IMT-P8-KLA. (Cells only: Black; KLA: blue and IMT-P8: Red). Overnight grown HeLa, and MDA-MB-231 cells were incubated with 2.5 μM FITC labeled peptides for 30 min and PC3 cells were incubated with 5 μM peptides for 1 h in serum free medium. After the incubation, cells were washed with PBS, rinsed with heparin (100 μg/ml), and then treated with trypsin (1 mg/ml) at 37 °C for 10 min. Finally cells were suspended in PBS, and subjected to flow cytometry. Bar diagrams showing the uptake of KLA and IMT-P8-KLA as mean cellular fluorescence in (**B**) HeLa, (**D**) MDA-MB-231 and (**F**) PC3 cells from the flow cytometric analysis of all live cells positive for FITC. In **B**,**D**, and **F**, data are expressed as mean ± S.E. based on triplicates of at least two independent experiments. Asterisks indicate significance according to student’s t-test (two-tailed); (*p < 0.05).

In order to examine the type of cargo IMT-P8 could deliver, first, KLA, a pro-apoptotic peptide, was conjugated to IMT-P8. Internalization of FITC-labeled IMT-P8-KLA in various human tumor cells (HeLa, MDA-MB-231 and PC3 cells) was monitored by incubating the cells with IMT-P8-KLA and KLA alone (2.5 μM) for 30 min at 37 °C followed by flow cytometry analysis. Since flow cytometry does not differentiate between the fluorescence obtained from internalized peptide and surface bound peptides; therefore, after treatment with FITC labeled peptides, cells were rinsed with heparin (100 μg/ml) and incubated with trypsin (1 mg/ml) for 10 min at 37 °C to remove the extracellular membrane-associated peptides^36^. The results of flow cytometry analysis are shown in Fig. 1. A significant intracellular FITC fluorescence was observed in HeLa (Fig. 1A,B), MDA-MB-231 (Fig. 1C,D) and PC3 cells (Fig. 1E,F) incubated with IMT-P8-KLA. In contrast, negligible fluorescence was observed in the case of cells treated with FITC-KLA peptide (Fig. 1). These results suggested that IMT-P8 efficiently delivered KLA peptide, which cannot internalize by its own, into the cancer cells.

**Figure 2:**
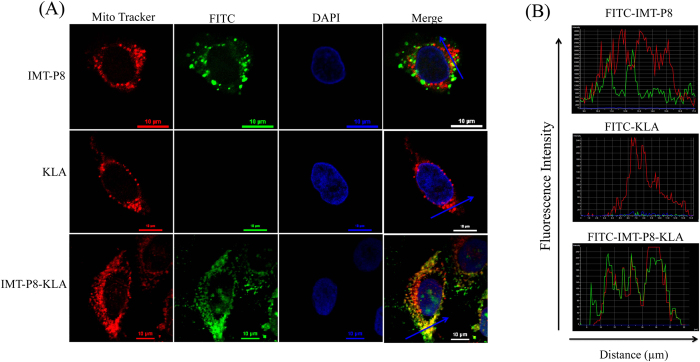
Intracellular localization of FITC-labeled peptides in HeLa cells. (**A**) Confocal images showing co-localization between FITC-labeled IMT-P8-KLA and Mito Tracker Red. Overnight grown HeLa cells were incubated both with FITC-IMT-P8 (2.5 μM) and Mito Tracker Red (100 nM) for 30 min at 37 °C. Cells were washed with PBS thrice and live cells were imaged. Scale bars are 10 *μm*. The laser intensity and photomultiplier settings have been adjusted to yield the best visualization of each image, and the intensities are thus not directly comparable. (**B**) Images showing the overlapping intensity spectra of FITC (green) and Mito tracker dye (red) along the blue line in merged image in (**A**).

**Figure 3:**
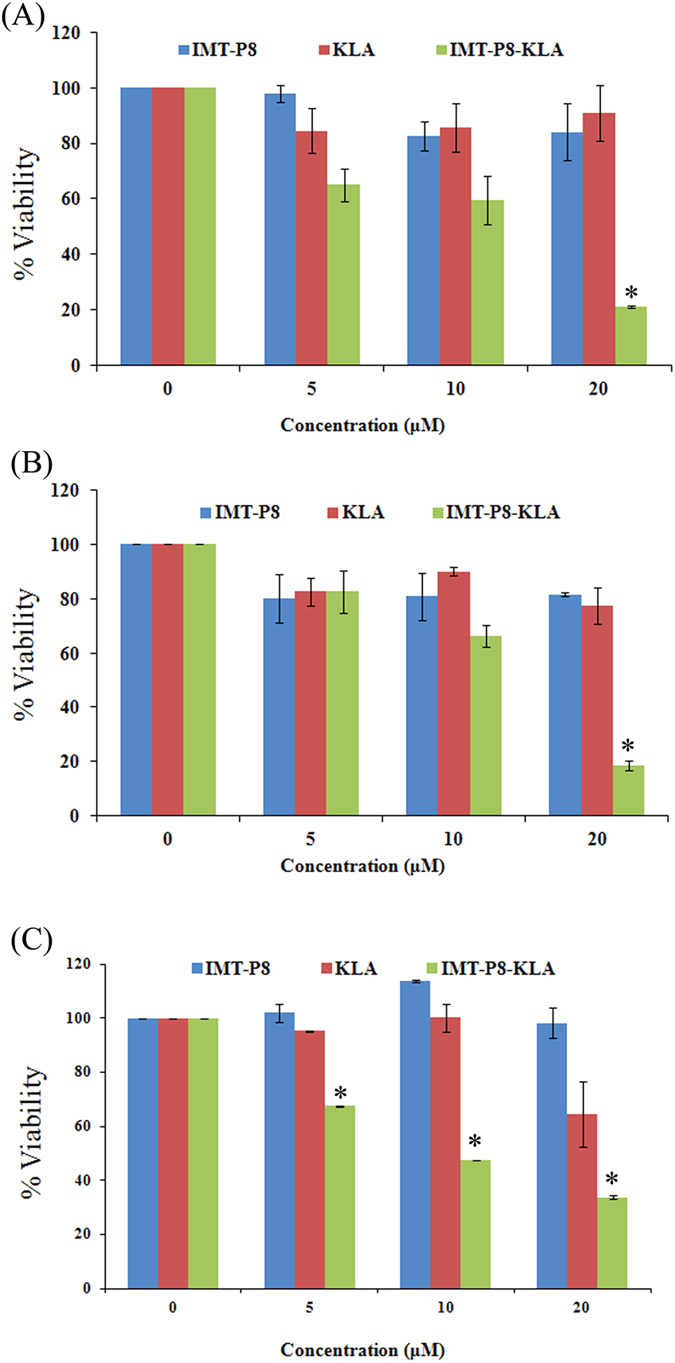
Cell viability assay. (**A**) HeLa, (**B**) PC3 and (**C**) MDA-MB-231 cells were incubated with increasing concentrations (5, 10, and 20 μM) of peptides in serum containing medium at 37 °C for 24 h. Cell viability was measured by MTT assay. Viability of only cells (without peptide) was taken as 100% and viabilities of cells treated with increasing concentration of peptides were plotted as percentage of control. Results are expressed as mean ± S.E. based on triplicates of at least two independent experiments. Asterisks indicate significance according to student’s t-test (two-tailed); (*p < 0.05).

Since KLA is a cationic amphipathic peptide and has been previously reported to accumulate in mitochondria^37^, we next examined the intracellular localization of IMT-P8-KLA. For this, HeLa cells treated with IMT-P8-KLA (2.5 μM) were analyzed by confocal-laser scanning microscopy (CLSM). As shown in Fig. 2A, IMT-P8-KLA internalized into the HeLa cells as a complete cytosolic fluorescence was observed in HeLa cells while no fluorescence was observed in the cells treated with KLA alone. These results were consistent with the fluorescence-activated cell sorting (FACS) analysis. CLSM analysis of cells treated with FITC-IMT-P8-KLA revealed its co-localization with MitoTracker, a mitochondria-specific dye (Fig. 2B) suggesting the accumulation of IMT-P8-KLA into the mitochondria. In contrast, under the same condition, FITC-IMT-P8 did not show any co-localization with Mitotracker dye (Fig. 2B) suggesting the specific mitochondrial targeting ability of IMT-P8-KLA. Since KLA is well known pro-apoptotic peptide that induces apoptosis of the cells by disrupting mitochondrial membrane, next we sought to examine the functional activity of IMT-P8-KLA inside the cells using 3-(4,5-dimethylthiazol-2-yl)-2,5-diphenyltetrazolium bromide (MTT) assay. For this, HeLa cells were incubated with increasing concentration (0, 5, 10 and 20 μM) of unlabeled IMT-P8-KLA and KLA alone for 24 h followed by determination of cell death using MTT assay. A dose-dependent decrease in cell viability was observed in all cell lines examined (Fig. 3). A significant decrease in cell viability was observed at 20 μM. All these results confirmed that IMT-P8 delivered KLA peptide into the cells and after internalization, IMT-P8-KLA localized to mitochondria where it causes cell death by disrupting the mitochondrial membrane.

### Purification of recombinant TAT-GFP and IMT-P8-GFP

**Figure 4:**
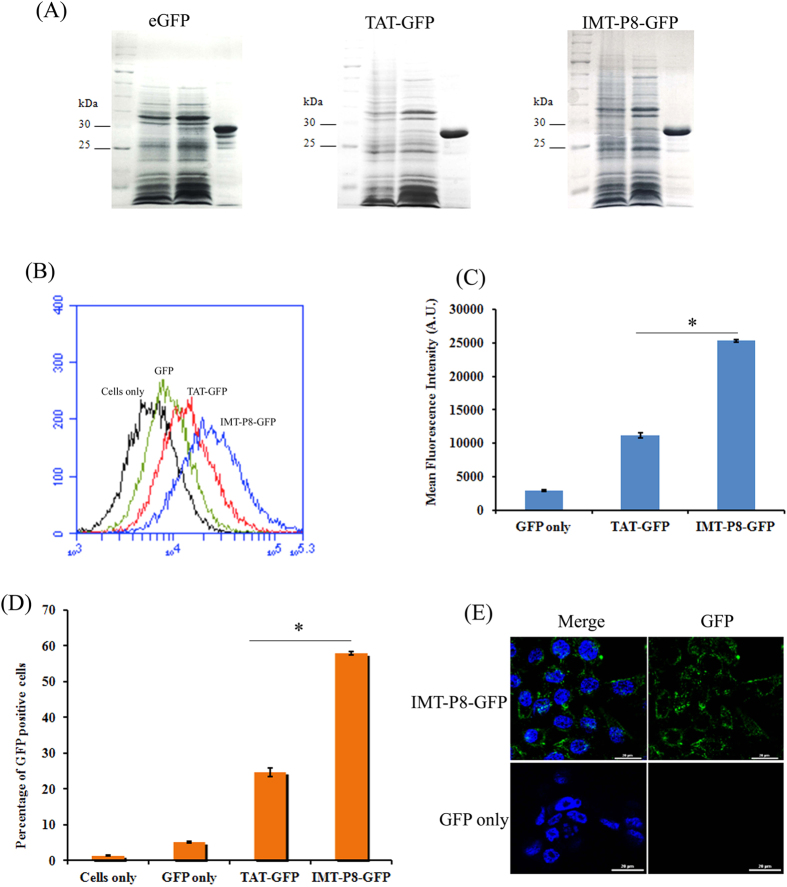
Characterization of recombinant proteins and cellular uptake of IMT-P8-GFP as determined by FACS analysis. (**A**) Identification of recombinant protein (GFP, TAT-GFP and IMT-P8-GFP) by coomassie brilliant blue staining. (**B**) Frequency distributions of GFP fluorescence intensity in HeLa cells incubated with GFP alone, TAT-GFP and IMT-P8-GFP (Cells only: black; GFP: green; TAT-GFP: red; and IMT-P8-GFP: blue). Overnight grown HeLa cells were incubated with 5 μM recombinant proteins for 1 h in serum free medium. After the incubation, cells were washed with PBS, rinsed with heparin (100 μg/ml), and then treated with trypsin (1 mg/ml) at 37 °C for 10 min. Finally cells were suspended in PBS, and subjected to flow cytometry. (**C**) Bar diagram showing the uptake of recombinant proteins as mean cellular fluorescence from the flow cytometric analysis of all live cells positive for GFP. (**D**) Bar diagram showing percentage of the GFP-positive cells. In **C**,**D**, results are expressed as mean ± S.E. based on triplicates of at least two independent experiments. Asterisks indicate significance according to student’s t-test (two-tailed); (*p < 0.05). (**E**) Intracellular localization of IMT-P8-GFP in HeLa cells as determined by confocal scanning laser microscope.

The GFP, TAT-GFP and IMT-P8-GFP-expressing vectors were constructed as explained in methods. For protein production and purification, *E. coli* BL21DE3star (pLysS) (Invitrogen) cells were transformed with the constructs mentioned above and recombinant proteins were expressed as soluble proteins. Recombinant proteins GFP (~27 kDa), TAT-GFP (~29 kDa), and IMT-P8-GFP (~29 kDa) were confirmed by Coomassie brilliant blue staining (Fig. 4A).

### *In vitro* delivery of GFP by IMT-P8

Next, to assess if IMT-P8 could carry large macromolecules such as GFP protein into the cells, internalization of IMT-P8-GFP was monitored by incubating HeLa cells with IMT-P8-GFP (5 μM) followed by flow cytometry analysis. Uptake of IMT-P8-GFP was compared with TAT-GFP and GFP alone. Before analysis, treated cells were rinsed with heparin (100 μg/ml) and incubated with trypsin (1 mg/ml) for 10 min to remove any membrane-associated protein^36^. As shown in Fig. 4B,C, a considerable higher GFP fluorescence intensity was observed in HeLa cells treated with IMT-P8-GFP compared to cells treated with TAT-GFP. Negligible fluorescence was observed in the cells treated with GFP alone. Similar observations were also observed when PC3 and RWPE-1 cells incubated with IMT-P8-GFP and TAT-GFP (data not shown). Approximately, 60% HeLa cells were found to be GFP-positive when treated with IMT-P8-GFP compared to cells treated with TAT-GFP in which only 25% cells were GFP-positive (Fig. 4D) suggesting that uptake of IMT-P8-GFP was efficient and higher than TAT-GFP.

To further confirm the internalization and to determine the intracellular localization of IMT-P8-GFP, HeLa cells were incubated with IMT-P8-GFP (5 μM) for 1 h at 37 °C and examined using confocal microscopy. As shown in Fig. 4E, IMT-P8-GFP internalized in the HeLa cells, which were consistent with the FACS analysis. A punctate pattern of IMT-P8-GFP fluorescence was observed indicating the presence of IMT-P8-GFP into the vesicular compartments after internalization (Fig. 4E). These results were similar to our previous observation with the IMT-P8 peptide that also showed a punctate fluorescence pattern and reminiscent of an endocytic mechanism of uptake^28^. Taken together all these results, it can be suggested that IMT-P8 delivered GFP more efficiently that TAT peptide into HeLa cells and IMT-P8-GFP was internalized through endocytosis.

### Skin penetrating properties of IMT-P8 *in vivo*

**Figure 5:**
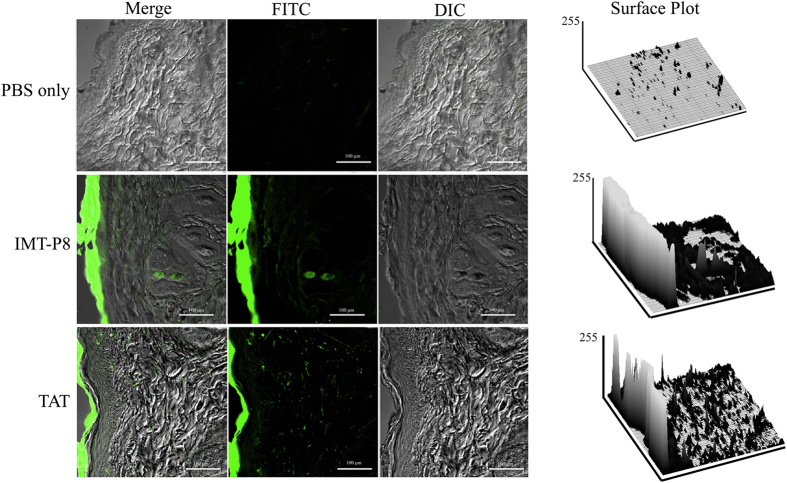
Penetration of IMT-P8 into mouse skin. Confocal images demonstrating the penetration of IMT-P8 in mice skin. Briefly, 15 μL of 1 mM peptides (FITC labeled TAT and IMT-P8) were topically applied onto a shaved area of mouse skin. Frozen vertical sections of skin tissues were obtained 2 h after the application of peptides and observed by a confocal laser-scanning microscope. Data are representative of three independent experiments with similar results.

Given the results demonstrating that IMT-P8 is capable of transporting KLA and GFP into human tumor cells, we wanted to investigate further whether IMT-P8 has the ability to penetrate SC, the outermost layer of the skin and enter into the epidermis, so that it could be used as carrier for topical and/or transdermal delivery of peptides and proteins. To address this, FITC-labeled IMT-P8 (15 μl of 1 mM) was applied topically to shaved mouse skin and the degree of skin penetration of IMT-P8 was examined 2 and 24 h after the topical application by analysis of the frozen skin sections under the confocal microscope. Since TAT has been used for topical delivery of cargoes in earlier studies^38^, FITC-TAT was also applied topically as a positive control while only PBS was used as the vehicle. As shown in Fig. 5, after 2 h, the skin sections treated with FITC-IMT-P8 and FITC-TAT revealed a significant green fluorescence throughout the epidermis and diffused fluorescence in the dermal tissue suggesting that IMT-P8 has the ability to penetrate SC and internalized into the epidermis. Along with epidermis, a clear green fluorescence in the hair follicles was also observed in the sections of skin treated with peptides (IMT-P8 and TAT) for 24 h suggesting the penetration of the peptides in the hair follicles (Supplementary Fig. S2). However, the intensity of green fluorescence was higher in the epidermis. No significant fluorescence was observed when only PBS was applied topically. Quantitation by compiling data from two-dimensional surface plots indicated that the FITC fluorescence intensity was 30.43 ± 6.46 and 18.733 ± 5.50 arbitrary units for FITC-IMT-P8 and FITC-TAT respectively (Supplementary Table S1). These results demonstrate that similar to TAT, IMT-P8 also has the ability to penetrate mouse skin.

### *In vivo* skin delivery of GFP and KLA using IMT-P8

**Figure 6:**
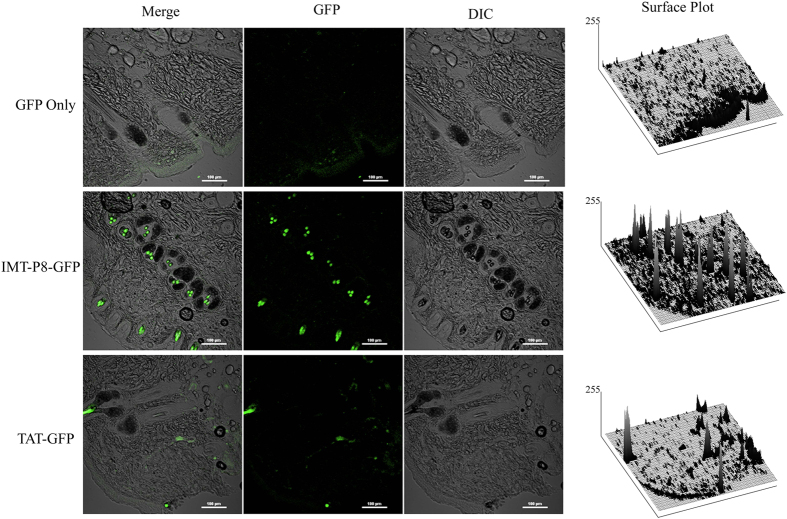
*In vivo* skin delivery of GFP using IMT-P8. Confocal images showing the internalization of IMT-P8-GFP in the mice skin. Mice skin was shaved one day before the start of the experiment. 30 μg of GFP, TAT-GFP and IMT-P8-GFP was applied to the shaved skin of mouse for 24 h. Frozen sections of skin tissues were obtained 24 h after the application of recombinant proteins and observed by a confocal laser scanning microscope.

The penetration ability of IMT-P8 into the mouse skin following topical application prompted us to investigate further the potential of delivery of GFP and KLA into the skin using IMT-P8. For this, IMT-P8-GFP, TAT-GFP and GFP (30 μg each) were applied topically on shaved skin of adult mice. The animals were sacrificed 24 h later, and frozen vertical sections of the treated region were examined by confocal microscopy. It was noted that in the case of GFP only, negligible green fluorescence was observed outside SC, in epidermis and dermis (Fig. 6), while a considerable high green fluorescence was observed in both epidermis, hair follicles, and in deeper layers of skin when treated with IMT-P8-GFP and TAT-GFP (Fig. 6). Quantitation by compiling data from two-dimensional surface plots indicated that GFP intensity was higher in the skin section treated with IMT-P8-GFP compared to TAT-GFP (Supplementary Table S1). In addition, IMT-P8-GFP fluorescence was localized to specific regions in the epidermis and dermis. These results suggested that IMT-P8-GFP and TAT-GFP were able to penetrate both the epidermis and dermis while GFP alone was not able to penetrate the skin.

**Figure 7:**
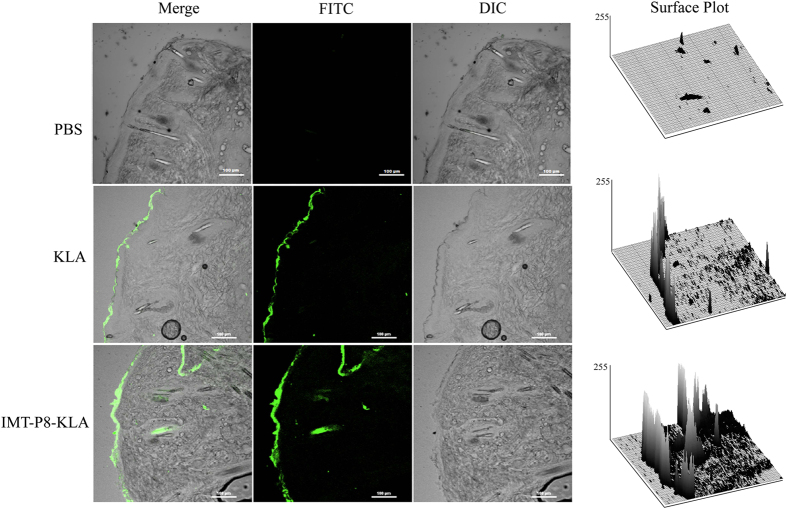
*In vivo* skin delivery of KLA using IMT-P8. Confocal images demonstrating the topical delivery of KLA using IMT-P8. 15 μL of 1 mM peptides (FITC labeled KLA and IMT-P8-KLA) were topically applied onto a shaved area of mouse skin. Frozen sections of skin tissues were obtained 2 h after the application of peptides and observed by a confocal laser-scanning microscope.

Similar findings were observed when FITC labeled IMT-P8-KLA and KLA (each 15 μL of 1 mM) were applied topically on mouse skin. After the 2 h incubation, a considerably higher FITC fluorescence was observed in the epidermis in the case of IMT-P8-KLA (Supplementary Table S1) whereas no significant fluorescence was observed in the epidermis in case of KLA alone (Fig. 7). A significant fluorescence was observed inside the hair follicles after 24 h suggesting the penetration of IMT-P8 KLA into the hair follicles (Supplementary Fig. S3). Little fluorescence was seen inside the hair follicles in the case of KLA. Skin treated with PBS alone showed no fluorescence. All these results demonstrated that IMT-P8 not only penetrates the SC but also is capable of transporting macromolecules like GFP and KLA into the skin.

## Discussion

Over the last decade, small peptides have emerged as potential alternatives to treat various diseases, including diabetes, oncology, and metabolic, cardiovascular, and infectious diseases^39^,^40^,^41^. The growth in the development of various therapeutic peptide databases^13^,^42^,^43^,^44^,^45^,^46^,^47^,^48^,^49^, as well as the *in silico* algorithms for the prediction of important properties of peptide like toxicity^50^,^51^, half-life^52^, antigenicity^53^,^54^,^55^,^56^, etc has expedited the peptide-based research. Among the various classes of therapeutic peptides, CPPs have garnered tremendous scientific attention as attractive drug delivery vehicle^41^,^57^. The CPP, IMT-P8 is an arginine-rich human protein-derived novel cell-penetrating peptide, which was discovered and characterized recently^28^ using integrated *in silico*^29^ and experimental approach. However, the cargo delivery capability of IMT-P8 had not been investigated previously. Therefore, the aim of the present study was to determine the cargo delivery capability of IMT-P8. In this context, first, we have examined the cargo delivery ability of IMT-P8 in various human tumor cell lines, including HeLa and PC3 cells.

We examined whether IMT-P8 can deliver small cargoes like a small peptide. Therefore, we selected KLA peptide as cargo, which is a small cationic amphipathic mitochondrial membrane-disrupting peptide, which causes mitochondrial membrane swelling leading to apoptosis when delivered inside the cells^58^,^59^. However, KLA cannot disturb plasma membrane and thus it is not toxic to eukaryotic cells. But when conjugated to CPPs, KLA becomes toxic because it can cross the plasma membrane and disrupt the negatively charged mitochondrial membrane and release cytochrome C^59^. Therefore, we have also examined this possibility whether IMT-P8 can deliver KLA inside the eukaryotic cells and cause cell death. Flow cytometry analysis of HeLa cells incubated with FITC-IMT-P8-KLA fusion peptide revealed the efficient internalization of IMT-P8-KLA into the HeLa cells, and these results were further confirmed by confocal microscopic analysis. As expected, KLA alone did not internalize into the cells. Interestingly, after internalization, IMT-P8-KLA got distributed throughout the cytoplasm and nucleus. Also, a major proportion of IMT-P8-KLA was localized to mitochondria. These results were consistent with the previous study where CPP-KLA was shown to accumulate into the mitochondria^60^. In contrast, the intracellular distribution of FITC-IMT-P8 was altogether different than FITC-IMT-P8-KLA and it did not localize to mitochondria. A punctate cytoplasmic pattern of FITC fluorescence was observed when HeLa cells were treated with FITC-IMT-P8 peptide suggesting that significant portion of IMT-P8 was entrapped into the vesicular compartments and this observation was in agreement with the previous findings that IMT-P8 internalized by endocytosis^28^. These results suggested that both IMT-P8 and IMT-P8-KLA internalized by endocytosis and after internalization, IMT-P8 got entrapped in the endosomes while IMT-P8-KLA could escape from the endosomes by perturbing the endosomal membrane and subsequently entered into nucleus and mitochondria. KLA is mitochondrial-disrupting peptide and causes cell death by mitochondrial membrane disruption^37^. Therefore, in order to ascertain the functionality of IMT-P8-KLA inside the cells, we examined the cell viability of HeLa cells treated with IMT-P8-KLA after 24 h incubation. IMT-P8-KLA caused significant cell death whereas both IMT-P8 and KLA alone were not effective at any concentration tested. All these results suggested that IMT-P8 delivered KLA successfully inside the human tumor cells, and the internalized peptide was functional.

Next, in order to determine whether IMT-P8 can translocate a large molecule, we chose GFP as cargo. GFP does not have the ability to internalize into the cell on its own, and it is endogenously fluorescent; therefore, the *in vitro* and *in vivo* localization can easily be monitored. Flow cytometry and confocal studies with HeLa (Fig. 4E), RWPE-1 and PC3 cells (data not shown) treated with IMT-P8-GFP confirmed that IMT-P8 is capable of delivering GFP into the cells even after 30 min as a significant GFP fluorescence was observed in these cell lines. In the previous study, uptake of IMT-P8 was found to be significantly higher than TAT, a widely used CPP. Here also, uptake of IMT-P8-GFP was significantly higher than TAT-GFP. The uptake mechanism of IMT-P8-GFP was supposed to be similar to IMT-P8 as a punctate cytoplasmic fluorescence was observed in HeLa cells treated with both IMT-P8-GFP and IMT-P8. Taken together all these results, it can be concluded that IMT-P8 is capable of transporting cargoes of different sizes ranging from small molecules like FITC and small peptides to large macromolecules.

Since peptides are unstable in the gastrointestinal tract and thus cannot be administered by oral route^40^. Therefore, most CPP-based formulations can be administered by intravenous route only, which is invasive and thus it is not beneficial to patients particularly when repetitive doses are required. Ideally, a drug delivery system should be non-invasive, painless, easy to use and provides better patient compliance. In this context, the transdermal delivery route has garnered tremendous attention over the last few years. Transdermal delivery of therapeutic molecules offers numerous advantages over the conventional oral delivery and injection. Though skin delivery has tremendous potential because of large surface area and easy accessibility, it is very challenging as skin serves as self-protection barrier and blocks the penetration of the extraneous molecules. Skin tissue consists of the four different layers: stratum corneum, viable epidermis, dermis and subcutaneous connective tissue^11^. Among these, the outermost layer is SC, which protects the entry of external materials into the skin. In the recent past, considerable efforts have been made to investigate the CPP-mediated transdermal delivery of therapeutic molecules like proteins^33^, nanoparticles^61^, hyaluronic acid^62^, small molecule drugs^27^, siRNA^26^, insulin^24^, etc. In these studies, CPPs have been found to enhance the permeation of therapeutic molecules by the local topical administration in the skin for treating various diseases, including psoriasis and atopic dermatitis.

Given the results demonstrating that IMT-P8 can deliver cargoes into the cells *in vitro*, next we sought to examine whether IMT-P8 is able to penetrate the mouse skin and deliver the cargoes into the skin layers *in vivo*. After 2 h of topical application, IMT-P8 crossed the SC, and accumulated into the epidermis. Little accumulation in dermal tissue was also observed suggesting that IMT-P8 can penetrate the skin (Fig. 5). After 24 h, along with accumulation in the epidermis, a strong green fluorescence was observed inside the hair follicles indicating the presence of IMT-P8 inside the hair follicles (Supplementary Fig. S2). Similar observation was seen when fusion molecules, IMT-P8-GFP and IMT-P8-KLA were applied topically suggesting that IMT-P8 capable of delivering protein and peptide cargoes into the skin. Earlier, Johnson LN *et al.*^63^ have reported the topical delivery of GFP using POD peptide on mice skin. They have shown that after topical application, POD-GFP was accumulated in the epidermis and inside the hair follicles. Our results were in close agreement with these previous findings.

The mechanism by which IMT-P8 internalized into the skin is not clear yet. But it seems that the mechanism for the skin penetration of IMT-P8 is somehow different from the plasma membrane penetration. IMT-P8 internalized cancer cells through macropinocytosis but in the case of skin, the SC is composed of non-viable cells and thus endocytosis is not expected. In the past, many studies have been performed on the use of small peptides to deliver cargoes into the skin^21^,^33^,^34^,^35^. However, the exact mechanism of peptide transport through skin layers is not fully understood. It has been suggested that arginine-rich CPPs like TAT and oligoarginine have a net high positive charge due to the arginine. After interaction with the negatively charged skin cell surfaces, they are transported through various skin layers using three major routes including transcellular, appendage, and intracellular (extracellular matrix) routes^11^. In the past, a few studies have been carried out reporting the localization/ route of small peptide penetration through skin layers. Chen Y *et al.*^24^ have shown the delivery of insulin by TD-1 peptide via skin. In this study, authors have shown the accumulation of FITC labeled-insulin inside the hair follicles following topical application suggesting the involvement of transfollicular route in the insulin delivery using TD-1 peptide. In a different study, Johnson LN *et al.*^63^ have also shown the penetration of POD-GFP into the hair follicles. In the present study, we have also observed the accumulation of IMT-P8 into the epidermis and hair follicles as a significant green fluorescence was observed in the epidermis and inside the hair follicles in the skin samples treated with IMT-P8, IMT-P8-KLA and IMT-P8-GFP. Therefore, based on these results as well as on the basis of previous research findings from other studies, it can be suggested that IMT-P8 may penetrate into the skin layers using transfollicular route. However, further studies are required to understand the exact mechanism(s) of the IMT-P8-mediated transport into the skin.

In summary, the present investigation demonstrates that IMT-P8 can transport a variety of cargoes into different cancer cell lines more efficiently than the TAT peptide. Apart from the *in vitro* cell-penetrating capability, IMT-P8 is also able to penetrate the skin tissue *in vivo* and can deliver a range of molecules from FITC to KLA to GFP into the mouse skin, which makes IMT-P8 a potential lead molecule to be used in the treatment of various local skin infections and different cosmetics applications.

## Methods

### Materials

Human cervical cancer cell line HeLa, human prostate carcinoma cell line PC3, human normal prostate epithelium cell line RWPE-1 and breast cancer cell line MDA-MB-231 were all obtained from American Type Culture Collection (ATCC; Manassas, VA). Dulbecco’s Modified Eagle’s Media (DMEM), RPMI-1640, Ham’s F12 nutrient mix, Keratinocyte-SFM, fetal bovine serum (FBS), trypsin, penicillin, streptomycin, Opti-MEM, phosphate buffer solution (PBS, pH 7.4), MitoTracker Red CMXRos (marker for mitochondria), DAPI (marker for nucleus) and antifade reagent were purchased from Molecular Probes (Eugene, OR). MTT reagent and other reagents used for protein expression and purification were purchased from Sigma. BCA protein assay kit was purchased from Pierce. Chloramphenicol, kanamycin and lysozyme were purchased from Sigma. All chemicals used were of analytical grade.

### Peptide synthesis

The list of peptides used in the present study is summarized in Table 1. IMT-P8 was synthesized at peptide synthesizing facility at our Institute of Microbial Technology, Chandigarh, India while rest two peptides KLA and IMT-P8-KLA were purchased from GL Biochem (Shanghai, China). The IMT-P8 peptide was synthesized by solid phase peptide synthesis strategy using Fmoc (N-(9-fluronyl)-methoxycarbonyl) chemistry in 0.01 mmole scale on a Protein Technologies Inc, USA, PS-3 peptide synthesizer as described elsewhere^28^. Peptides were labeled with fluorescein isothiocyanate (FITC) using an amino hexanoic acid (ahx) linker at the N-terminus.

### Plasmid construction

The GFP coding region was amplified from the pEGFP-c1 plasmid (Clontech, USA) and cloned into the NdeI and BamHI sites of expression vector pET28a. TAT-GFP and IMT-P8-GFP were PCR-amplified and cloned into NdeI and XhoI site within the multi-cloning site of an expression vector, pET23a using oligonucleotide pairs (Supplementary Table S2). This resulted in full-length aforementioned proteins with a C-terminal His 6-tag. All constructions were verified by DNA sequencing.

### Recombinant protein purification

For protein production and purification, *E. coli* BL21DE3star (pLysS) (Invitrogen) cells were transformed with the above-mentioned constructs and expressed as soluble proteins. The transformed cells were grown overnight, and this stock was used to inoculate further 1000 mL LB broth containing 50 μg/mL kanamycin and 34 μg/mL chloramphenicol. The cells were grown at 37 °C in an incubator shaker at 200 rpm to an A_600_ of 0.6 and protein expression was induced with 1 mM IPTG. They were further grown for 4 hrs at 37 °C and harvested by centrifugation. The cell pellet was resuspended in ice-cold buffer A (20 mM Na+ phosphate buffer, pH 7.2 and 300 mM NaCl) containing 0.1 mg/ml lysozyme and incubated on ice for 30 minutes. All subsequent procedures were performed at 4 °C. The cell suspension was sonicated, and the supernatant was collected after centrifugation at 11000 g for 60 min. Since the proteins of interest were either GFP/GFP-fusion constructs, it was possible to ascertain visually their presence in the various steps of the protocol. The supernatant was passed through equilibrated Ni-NTA agarose beads (3 ml of 50% slurry). This was followed by 5 column volumes of washing with buffer A containing 30 mM imidazole to remove non-specifically bound protein. The proteins were eluted in 10 mL buffer A containing 300 mM imidazole. To remove imidazole, the eluted proteins were added separately to Amicon protein concentrators, and the buffer was exchanged three times using buffer A alone (without imidazole). The proteins were finally concentrated 5-fold, and the volume brought down to 2 ml. These proteins were quantitated using BCA protein assay kit (Pierce). This protocol yielded ~3–4 mg protein each of GFP, TAT-GFP, and IMT-P8-GFP. Proteins were aliquoted and stored at −80 °C till further use.

### Cell culture

PC3 and MDA-MB-231cells were grown in RPMI medium supplemented with 10% FBS (Gibco) and L-glutamine (2 mM). HeLa cells were cultured in DMEM, supplemented with 10% FBS and 1% penicillin/streptomycin antibiotics (Gibco). RWPE-1 cells were cultured in Keratinocyte-SFM medium (Gibco) supplemented with the human recombinant epidermal growth factor (Gibco) and bovine pituitary extract (Gibco). All cells were maintained at 37 °C in humidified 5% CO_2_ atmosphere.

### Quantification of cellular uptake

To quantify the uptake of CPP-GFP fusion proteins (TAT-GFP, IMT-P8-GFP) and FITC-labeled peptides (KLA, and IMT-P8-KLA), HeLa cells (2.0 × 10^5^ per well) were seeded at 37 °C onto 24-well plates approximately 24 h before the start of experiments. Thereafter, cells were washed with PBS and incubated with either CPP-GFP fusion proteins (5 μM for 1 h) or FITC-labeled peptides (2.5 μM each for 30 min) in OptiMEM medium at 37 °C. Following the above treatment, cells were washed with PBS twice to remove the excess extracellular unbound peptides. Subsequently, cells were rinsed twice with heparin (100 μg/ml) and incubated with trypsin (1 mg/ml) for 10 min to remove non-internalized surface bound peptides or proteins. Following this, cells were centrifuged at 1000 rpm for 5 min, washed and finally suspended in PBS. FITC fluorescence intensity of internalized peptides or proteins in live cells was measured by flow cytometry using Accuri C6 flow cytometer (BD Biosciences) by acquiring 10,000 live cells. Experiments were carried out twice in triplicate. Data was obtained and analyzed using CFlow Sampler (BD Biosciences). The error bars indicate the standard error and untreated cells in media alone were used as controls.

### Confocal laser scanning microscopy

In order to visualize the distribution of internalized FITC labeled peptides and IMT-P8-GFP fusion proteins, HeLa cells (1 × 10^5^ cells) were seeded onto 12 well plates containing 16 mm glass coverslips, 24 h prior to incubation with FITC-labeled peptides or IMT-P8-GFP. After complete adhesion, the cell culture medium was replaced with fresh medium containing FITC labeled peptides (2.5 μM) and IMT-P8-GFP (5 μM), and then the cells were incubated at 37 °C for 30 min. Cells were not fixed to avoid artifactual localization of the internalized peptide. At the end of the incubation period, culture medium was removed, and coverslips were washed thoroughly with PBS for 3–5 minutes (thrice) and mounted on glass slides with the antifade reagent. Localization of FITC-labeled peptides and IMT-P8-GFP in the live cells (unfixed) was analyzed immediately using the Nikon A1R confocal microscope.

For co-localization studies, HeLa cells, grown on coverslips, were incubated with MitoTracker Red CMXRos (100 nM) along with FITC-IMT-P8 and FITC-IMT-P8-KLA (2.5 μM) in serum-free medium for 30 min at 37 °C. Thereafter, cells were washed with PBS thrice and immediately examined using the confocal microscope.

### Cell viability assay

Cell viability was determined by MTT assay. Briefly, cells (HeLa, PC3 and MDA-MB-231) were seeded at a density of 5 × 10^3^ cells/well in 96-well microtiter plates in medium supplemented with 10% FBS 24 h before the start of the experiment. Cells were incubated with different concentrations (5, 10, and 20 μM, respectively) of unlabeled peptides for 24 h. Control cells did not receive any peptide treatment. At the end of the incubation period, cell growth was assayed by the addition of 20 μl of MTT (5 mg/mL; Sigma-Aldrich) to each well, and plate was incubated at 37 °C for 4 h. Later, the medium was removed carefully, 200 μl DMSO was added and mixed, and the absorbance was read at 570 nm with a microplate reader (Bio-Rad). The survival of cells relative to the control (cells incubated with growth medium containing no peptide) was calculated by taking the ratio of the absorbance at 570 nm values. All the experiments were performed in triplicates.

### Animals and ethics statements

Male BALB/c mice of 6–8 weeks of age and weighing 22–25 g were obtained from the Animal Facility of Institute of Microbial Technology (IMTECH), Chandigarh. Mice were housed in individual cages under standard conditions (Temperature at 22 ± 2 °C, 12 hr light/ dark cycle) and were fed with normal pellet diet and water ad libitum. All the protocols on mice were approved by the Animal Ethics Committee of the IMTECH wide approval no. IAEC/13/21. All experiments on animals were performed as per the guidelines of the Committee for the Purpose of Supervision of Experiments on Animals (CPCSEA), national regulatory body for experiments on animals, Ministry of Environment & Forests, India. Proper care was taken to minimize pain and suffering of mice.

### *In vivo* skin delivery of KLA and GFP by IMT-P8

Topical delivery of GFP and KLA by IMT-P8 was evaluated on mice skin. The overall procedure is shown in Supplementary Fig. S1. Briefly, one day prior to topical application, a small region (2 × 2 cm) on the lateral sides of the abdomen of mice was shaved. Proper care was taken to prevent any damage to mouse skin while shaving. Next day, animals were anesthetized with xylazine/ketamine and 15 μl (stock 1 mM each) of FITC labeled IMT-P8, TAT, KLA, IMT-P8-KLA (n = 4) in PBS or 30 μg of each of the recombinant proteins (GFP, TAT-GFP, and IMT-P8-GFP) were applied to the shaved surface of abdominal skin (n = 6). GFP and PBS were applied to mouse skin as a control. In the case of peptides, the skin was harvested after 2 and 24 h whereas in the case of recombinant proteins it was harvested after 4, 12 and 24 h. However, the data was shown only for 24 h in the case of recombinant proteins. Before harvesting, skin was cleaned with 70% isopropyl alcohol just to remove the surface adsorbed peptide and immediately skin biopsies were fixed overnight in ice cold 4% formaldehyde. Thereafter, these skin samples were washed and immersed in 4.5% sucrose solution for 24 h followed by dehydration in 30% sucrose till deposition. Vertical cryosections (20 μm thickness) were obtained on a freezing microtome (Leica). Cryosections were mounted onto poly-L-lysine coated glass slides in 10 μl of mounting medium (Invitrogen). The skin sections were imaged on the confocal microscope (Nikon). For quantification of mean fluorescence, confocal images were analyzed using software ImageJ (http://ImageJ.nih.gov/ij/) developed by National Institutes of Health. Identical regions of each image were measured for each application. ImageJ converts image pixels into brightness values (arbitrary units). Surface plots were developed and brightness values of all the images were measured and represented as mean fluorescence.

### Statistical analysis

Statistical significance of the differences was determined by the two-tailed Student t-test using Microsoft Excel software. P values < 0.05 were considered as statistically significant.

## Additional Information

**How to cite this article**: Gautam, A. *et al.* Topical Delivery of Protein and Peptide Using Novel Cell Penetrating Peptide IMT-P8. *Sci. Rep.* **6**, 26278; doi: 10.1038/srep26278 (2016).

## Supplementary Material

Supplementary Information

## Figures and Tables

**Table 1:** List of peptides examined.

Peptide sequence*	Name	Length (aa)
RRWRRWNRFNRRRCR	IMT-P8	15
GRKKRRQRRRPPQ	TAT	13
KLAKLAKKLAKLAK	KLA	14
KLAKLAKKLAKLAKRRWRRWNRFNRRRCR	IMT-P8-KLA	29

^*^All peptides were FITC labeled at the N-terminus through amino hexanoic acid linker. The C-terminus of all peptides is free.

## References

[b1] Kim Y. C., Ludovice P. J. & Prausnitz M. R. Transdermal delivery enhanced by antimicrobial peptides. J Biomed Nanotechnol 6, 612–620 (2010). 21329054 10.1166/jbn.2010.1158

[b2] Wertz P. W. Lipids and barrier function of the skin. Acta Derm Venereol Suppl (Stockh) 208, 7–11 (2000). 10884933 10.1080/000155500750042790

[b3] Bos J. D. & Meinardi M. M. The 500 Dalton rule for the skin penetration of chemical compounds and drugs. Exp Dermatol 9, 165–169 (2000). 10839713 10.1034/j.1600-0625.2000.009003165.x

[b4] Prausnitz M. R. & Langer R. Transdermal drug delivery. Nat Biotechnol 26, 1261–1268 (2008). 18997767 10.1038/nbt.1504 PMC2700785

[b5] Prausnitz M. R., Mitragotri S. & Langer R. Current status and future potential of transdermal drug delivery. Nat Rev Drug Discov 3, 115–124 (2004). 15040576 10.1038/nrd1304

[b6] Karande P., Jain A. & Mitragotri S. Discovery of transdermal penetration enhancers by high-throughput screening. Nat Biotechnol 22, 192–197 (2004). 14704682 10.1038/nbt928

[b7] Kalia Y. N., Naik A., Garrison J. & Guy R. H. Iontophoretic drug delivery. Adv Drug Deliv Rev 56, 619–658 (2004). 15019750 10.1016/j.addr.2003.10.026

[b8] Williams A. C. & Barry B. W. Penetration enhancers. Adv Drug Deliv Rev 56, 603–618 (2004). 15019749 10.1016/j.addr.2003.10.025

[b9] Denet A. R., Vanbever R. & Preat V. Skin electroporation for transdermal and topical delivery. Adv Drug Deliv Rev 56, 659–674 (2004). 15019751 10.1016/j.addr.2003.10.027

[b10] Prausnitz M. R. Microneedles for transdermal drug delivery. Adv Drug Deliv Rev 56, 581–587 (2004). 15019747 10.1016/j.addr.2003.10.023

[b11] Nasrollahi S. A., Taghibiglou C., Azizi E. & Farboud E. S. Cell-penetrating peptides as a novel transdermal drug delivery system. Chem Biol Drug Des 80, 639–646 (2012). 22846609 10.1111/cbdd.12008

[b12] Kumar S. *et al.* Peptides as skin penetration enhancers: mechanisms of action. J Control Release 199, 168–178 (2015). 25499919 10.1016/j.jconrel.2014.12.006

[b13] Gautam A. *et al.* CPPsite: a curated database of cell penetrating peptides. Database (Oxford) 2012, 10.1093/database/bas015 (2012). PMC3296953 22403286

[b14] Milletti F. Cell-penetrating peptides: classes, origin, and current landscape. Drug Discov Today 17, 850–860 (2012). 22465171 10.1016/j.drudis.2012.03.002

[b15] Agrawal P. *et al.* CPPsite 2.0: a repository of experimentally validated cell-penetrating peptides. Nucleic Acids Res 44, D1098–1103 (2016). 26586798 10.1093/nar/gkv1266 PMC4702894

[b16] Gooding M., Browne L. P., Quinteiro F. M. & Selwood D. L. siRNA delivery: from lipids to cell-penetrating peptides and their mimics. Chem Biol Drug Des 80, 787–809 (2012). 22974319 10.1111/cbdd.12052

[b17] Nakase I., Tanaka G. & Futaki S. Cell-penetrating peptides (CPPs) as a vector for the delivery of siRNAs into cells. Mol Biosyst 9, 855–61 (2013). 23306408 10.1039/c2mb25467k

[b18] Nasrollahi S. A., Fouladdel S., Taghibiglou C., Azizi E. & Farboud E. S. A peptide carrier for the delivery of elastin into fibroblast cells. Int J Dermatol 51, 923–929 (2012). 22788807 10.1111/j.1365-4632.2011.05214.x

[b19] Liu S., Yang H., Wan L., Cheng J. & Lu X. Penetratin-Mediated Delivery Enhances the Antitumor Activity of the Cationic Antimicrobial Peptide Magainin II. Cancer Biother Radiopharm 28, 289–97 (2013). 23286306 10.1089/cbr.2012.1328

[b20] Kamada H. *et al.* Creation of novel cell-penetrating peptides for intracellular drug delivery using systematic phage display technology originated from Tat transduction domain. Biol Pharm Bull 30, 218–223 (2007). 17268054 10.1248/bpb.30.218

[b21] Rothbard J. B. *et al.* Conjugation of arginine oligomers to cyclosporin A facilitates topical delivery and inhibition of inflammation. Nat Med 6, 1253–1257 (2000). 11062537 10.1038/81359

[b22] Kim Y. C., Ludovice P. J. & Prausnitz M. R. Transdermal delivery enhanced by magainin pore-forming peptide. J Control Release 122, 375–383 (2007). 17628164 10.1016/j.jconrel.2007.05.031 PMC2035950

[b23] Lee J., Jung E., Park J. & Park D. Transdermal delivery of interferon-gamma (IFN-gamma) mediated by penetratin, a cell-permeable peptide. Biotechnol Appl Biochem 42, 169–173 (2005). 15804234 10.1042/BA20050003

[b24] Chen Y. *et al.* Transdermal protein delivery by a coadministered peptide identified via phage display. Nat Biotechnol 24, 455–460 (2006). 16565728 10.1038/nbt1193

[b25] Hsu T. & Mitragotri S. Delivery of siRNA and other macromolecules into skin and cells using a peptide enhancer. Proc Natl Acad Sci USA 108, 15816–15821 (2011). 21903933 10.1073/pnas.1016152108 PMC3179050

[b26] Chen M. *et al.* Topical delivery of siRNA into skin using SPACE-peptide carriers. J Control Release 179, 33–41 (2014). 24434423 10.1016/j.jconrel.2014.01.006 PMC4425738

[b27] Chen M. *et al.* Topical delivery of Cyclosporine A into the skin using SPACE-peptide. J Control Release 199, 190–197 (2015). 25481447 10.1016/j.jconrel.2014.11.015

[b28] Gautam A. *et al.* Identification and characterization of novel protein-derived arginine-rich cell-penetrating peptides. Eur J Pharm Biopharm 89, 93–106 (2015). 25459448 10.1016/j.ejpb.2014.11.020

[b29] Gautam A. *et al.* *In silico* approaches for designing highly effective cell penetrating peptides. J Transl Med 11, 74, 10.1186/1479-5876-11-74 (2013). 23517638 PMC3615965

[b30] Zhao M. & Weissleder R. Intracellular cargo delivery using tat peptide and derivatives. Med Res Rev 24, 1–12 (2004). 14595670 10.1002/med.10056

[b31] Saalik P. *et al.* Protein cargo delivery properties of cell-penetrating peptides. A comparative study. Bioconjug Chem 15, 1246–1253 (2004). 15546190 10.1021/bc049938y

[b32] Hitsuda T. *et al.* A protein transduction method using oligo-arginine (3R) for the delivery of transcription factors into cell nuclei. Biomaterials 33, 4665–4672 (2012). 22465335 10.1016/j.biomaterials.2012.02.049

[b33] Hou Y. W. *et al.* Transdermal delivery of proteins mediated by non-covalently associated arginine-rich intracellular delivery peptides. Exp Dermatol 16, 999–1006 (2007). 18031459 10.1111/j.1600-0625.2007.00622.x

[b34] Cohen-Avrahami M., Libster D., Aserin A. & Garti N. Penetratin-induced transdermal delivery from H(II) mesophases of sodium diclofenac. J Control Release 159, 419–428 (2012). 22306174 10.1016/j.jconrel.2012.01.025

[b35] Manosroi J. *et al.* Transdermal absorption and stability enhancement of salmon calcitonin by Tat peptide. Drug Dev Ind Pharm 39, 520–525 (2013). 22564052 10.3109/03639045.2012.684388

[b36] Richard J. P. *et al.* Cell-penetrating peptides. A reevaluation of the mechanism of cellular uptake. J Biol Chem 278, 585–590 (2003). 12411431 10.1074/jbc.M209548200

[b37] Law B., Quinti L., Choi Y., Weissleder R. & Tung C. H. A mitochondrial targeted fusion peptide exhibits remarkable cytotoxicity. Mol Cancer Ther 5, 1944–1949 (2006). 16928814 10.1158/1535-7163.MCT-05-0509

[b38] Lohcharoenkal W. *et al.* Potent enhancement of GFP uptake into HT-29 cells and rat skin permeation by coincubation with tat peptide. J Pharm Sci 100, 4766–4773 (2011). 21681754 10.1002/jps.22671

[b39] Kaspar A. A. & Reichert J. M. Future directions for peptide therapeutics development. Drug Discov Today 18, 807–817 (2013). 23726889 10.1016/j.drudis.2013.05.011

[b40] Vlieghe P., Lisowski V., Martinez J. & Khrestchatisky M. Synthetic therapeutic peptides: science and market. Drug Discov Today 15, 40–56 (2010). 19879957 10.1016/j.drudis.2009.10.009

[b41] Fonseca S. B., Pereira M. P. & Kelley S. O. Recent advances in the use of cell-penetrating peptides for medical and biological applications. Adv Drug Deliv Rev 61, 953–964 (2009). 19538995 10.1016/j.addr.2009.06.001

[b42] Kapoor P. *et al.* TumorHoPe: a database of tumor homing peptides. PLos One 7, e35187, 10.1371/journal.pone.0035187 (2012). 22523575 PMC3327652

[b43] Gautam A. *et al.* Hemolytik: a database of experimentally determined hemolytic and non-hemolytic peptides. Nucleic Acids Res 42, D444–449 (2014). 24174543 10.1093/nar/gkt1008 PMC3964980

[b44] Mehta D. *et al.* ParaPep: a web resource for experimentally validated antiparasitic peptide sequences and their structures. Database (Oxford) 2014, 10.1093/database/bau051 (2014). PMC4054663 24923818

[b45] Tyagi A. *et al.* CancerPPD: a database of anticancer peptides and proteins. Nucleic Acids Res 43, D837–843 (2015). 25270878 10.1093/nar/gku892 PMC4384006

[b46] Kumar R. *et al.* AHTPDB: a comprehensive platform for analysis and presentation of antihypertensive peptides. Nucleic Acids Res 43, D956–962 (2015). 25392419 10.1093/nar/gku1141 PMC4383949

[b47] Van Dorpe S. *et al.* Brainpeps: the blood-brain barrier peptide database. Brain Struct Funct 217, 687–718 (2012). 22205159 10.1007/s00429-011-0375-0

[b48] Wynendaele E. *et al.* Quorumpeps database: chemical space, microbial origin and functionality of quorum sensing peptides. Nucleic Acids Res 41, D655–659 (2013). 23180797 10.1093/nar/gks1137 PMC3531179

[b49] Di Luca M., Maccari G., Maisetta G. & Batoni G. BaAMPs: the database of biofilm-active antimicrobial peptides. Biofouling 31, 193–199 (2015). 25760404 10.1080/08927014.2015.1021340

[b50] Gupta S. *et al.* Peptide toxicity prediction. Methods Mol Biol 1268, 143–157 (2015). 25555724 10.1007/978-1-4939-2285-7_7

[b51] Gupta S. *et al.* *In silico* approach for predicting toxicity of peptides and proteins. PLos One 8, e73957, 10.1371/journal.pone.0073957 (2013). 24058508 PMC3772798

[b52] Sharma A., Singla D., Rashid M. & Raghava G. P. Designing of peptides with desired half-life in intestine-like environment. BMC Bioinformatics 15, 282, 10.1186/1471-2105-15-282 (2014). 25141912 PMC4150950

[b53] Dhanda S. K., Gupta S., Vir P. & Raghava G. P. Prediction of IL4 inducing peptides. Clin Dev Immunol 2013, 263952, 10.1155/2013/263952 (2013). 24489573 PMC3893860

[b54] Gupta S., Ansari H. R., Gautam A., Open Source Drug Discovery, C. & Raghava G. P. Identification of B-cell epitopes in an antigen for inducing specific class of antibodies. Biol Direct 8, 27, 10.1186/1745-6150-8-27 (2013). 24168386 PMC3831251

[b55] Singh H., Ansari H. R. & Raghava G. P. Improved method for linear B-cell epitope prediction using antigen’s primary sequence. PLos One 8, e62216, 10.1371/journal.pone.0062216 (2013). 23667458 PMC3646881

[b56] Ansari H. R. & Raghava G. P. *In silico* models for B-cell epitope recognition and signaling. Methods Mol Biol 993, 129–138 (2013). 23568468 10.1007/978-1-62703-342-8_9

[b57] Heitz F., Morris M. C. & Divita G. Twenty years of cell-penetrating peptides: from molecular mechanisms to therapeutics. Br J Pharmacol 157, 195–206 (2009). 19309362 10.1111/j.1476-5381.2009.00057.x PMC2697800

[b58] Chen W. H. *et al.* Dual-targeting pro-apoptotic peptide for programmed cancer cell death via specific mitochondria damage. Sci Rep 3, 3468, 10.1038/srep03468 (2013). 24336626 PMC3863817

[b59] Fu B. *et al.* Enhanced antitumor effects of the BRBP1 compound peptide BRBP1-TAT-KLA on human brain metastatic breast cancer. Sci Rep 5, 8029, 10.1038/srep08029 (2015). 25619721 PMC4306141

[b60] Nakase I. *et al.* Transformation of an antimicrobial peptide into a plasma membrane-permeable, mitochondria-targeted peptide via the substitution of lysine with arginine. Chem Commun (Camb) 48, 11097–11099 (2012). 23038156 10.1039/c2cc35872g

[b61] Desai P., Patlolla R. R. & Singh M. Interaction of nanoparticles and cell-penetrating peptides with skin for transdermal drug delivery. Mol Membr Biol 27, 247–259 (2010). 21028936 10.3109/09687688.2010.522203 PMC3061229

[b62] Chen M., Gupta V., Anselmo A. C., Muraski J. A. & Mitragotri S. Topical delivery of hyaluronic acid into skin using SPACE-peptide carriers. J Control Release 173, 67–74 (2014). 24129342 10.1016/j.jconrel.2013.10.007 PMC4128962

[b63] Johnson L. N., Cashman S. M., Read S. P. & Kumar-Singh R. Cell penetrating peptide POD mediates delivery of recombinant proteins to retina, cornea and skin. Vision Res 50, 686–697 (2010). 19733192 10.1016/j.visres.2009.08.028 PMC2840056

