# Supplementary material for: Topical Delivery of Protein and Peptide Using Novel Cell Penetrating Peptide IMT-P8
Source: Sci Rep. 2016 May 18;6:26278. doi: 10.1038/srep26278 (PMC4870705; doi:10.1038/srep26278)

Topical Delivery of Protein and Peptide Using Novel Cell Penetrating Peptide IMT-P8

Ankur Gautam[1*], Jagpreet Singh Nanda[1], Jesse S. Samuel[1], Manisha Kumari[1], Priyanka Priyanka[1], Gursimran Bedi[1], Samir K. Nath[2], Garima Mittal[3], Neeraj Khatri [3] and Gajendra Pal Singh Raghava[1*]

[1]Bioinformatics Centre, CSIR-Institute of Microbial Technology, Chandigarh-160036, India

[2]Department of Protein Science and Engineering, CSIR-Institute of Microbial Technology, Chandigarh-160036, India

[3]Experimental Animal Facility, CSIR-Institute of Microbial Technology, Chandigarh-160036, India

* Co-corresponding authors

G. P. S. Raghava, Bioinformatics Centre, CSIR-Institute of Microbial Technology, Chandigarh-160036, India, Phone: +91-172-2690557, Fax: +91-172-2690632
E.mail: raghava@imtech.res.in

Ankur Gautam, Bioinformatics Centre, CSIR-Institute of Microbial Technology, Chandigarh-160036, India, Phone: +91-172-2690557, Fax: +91-172-2690632
Email: ankurgautam@imtech.res.in

Supplementary Information

Table S1: Mean fluorescence of images computed using software ImageJ.

Treatment	Mean Fluorescence Pixel Intensity (AU)
**Peptide labeled with FITC**	
PBS only	1.139±0.107
IMT-P8-FITC	30.43±6.46
TAT-FITC	18.733±5.50
**Fusion peptide (KLA)**	
PBS only	1.004±0.010
KLA-FITC	3.50±0.51
IMT-P8-KLA-FITC	8.842±3.061
**Recombinant protein (GFP)**	
GFP only	1.66±0.412
IMT-P8-GFP	5.081±0.50
TAT-GFP	3.29±1.05

Table S2. List of primers used.

Primer name	Sequence (5’-3’)
IMT-P8-GFP-F	ATCATATACCATGGGAAGAAGATGGAGAAGATGGAACAGAT TCAACAGAAGAAGATGCAGAATGGTGAGCAAGGGCGAGGAG
IMT-P8-GFP-R	ATGTCACTCGAGCTTGTACAGCTCGTCCATGCC
TAT-GFP-F	TTACGACCATGGGCGGAAGAAAAAAAAGAAGACAAAGAAGA AGACCACCACAA ATGGTGAGCAAGGGCGAG
TAT-GFP-R	ATGTCACTCGAGCTTGTACAGCTCGTCCATGCC

Figure S1. Schematic representation of procedure for in vivo topical delivery of GFP and KLA using IMT-P8.

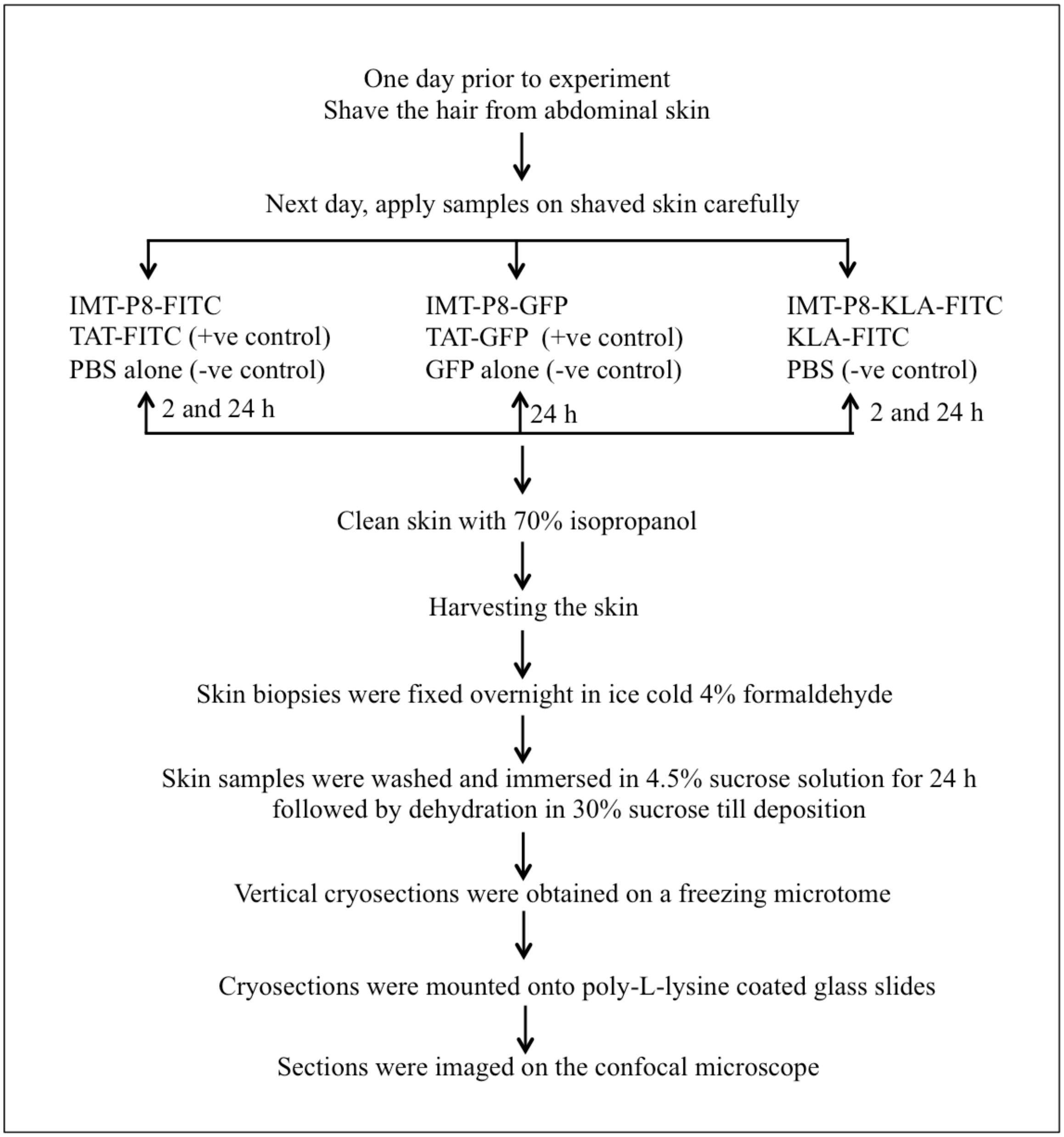

Figure S2. *In vivo* topical delivery of IMT-P8. Confocal images demonstrating the penetration of IMT-P8 in mice skin. Briefly, 15 μL of 1 mM peptides (FITC-labeled peptides TAT and IMT-P8) were topically applied onto a shaved area of mouse skin. Frozen vertical sections of skin tissues were obtained 24 h after the application of peptides and observed by a confocal laser-scanning microscope.

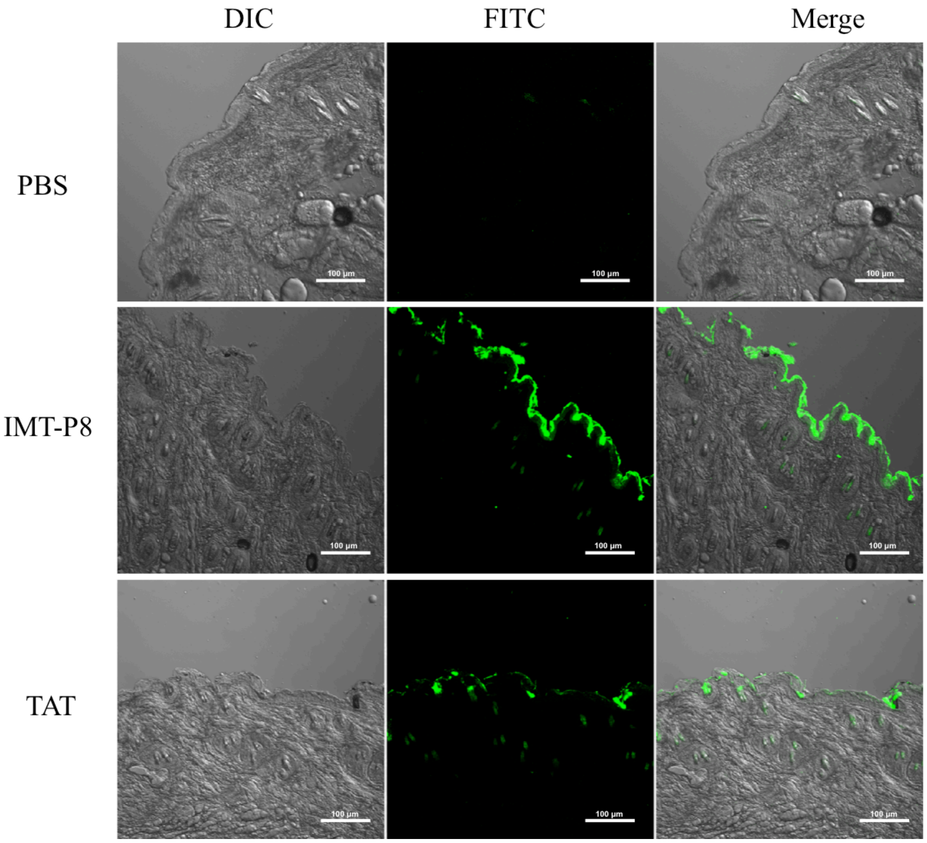

Figure S3. ***In vivo*** **topical delivery of IMT-P8-KLA.** Confocal images demonstrating the penetration of IMT-P8-KLA in mice skin. Briefly, 15 μL of 1 mM peptides (FITC-labeled peptides KLA and IMT-P8-KLA) were topically applied onto a shaved area of mouse skin. Frozen vertical sections of skin tissues were obtained 24 h after the application of peptides and observed by a confocal laser-scanning microscope.

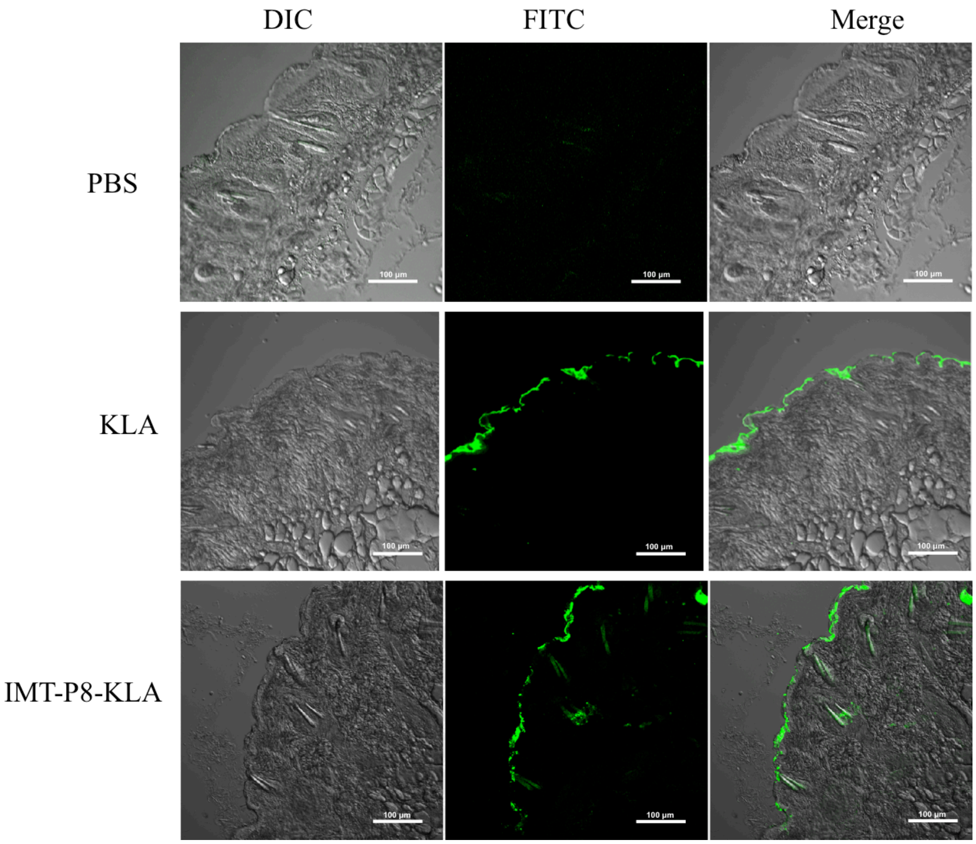

Supplement: Supplementary Information [file srep26278-s1.pdf]
